# Readmission Rate, Predictors, Outcomes, and Burden of Readmission of Hepatorenal Syndrome in the United States: A Nationwide Analysis

**DOI:** 10.1002/jgh3.70062

**Published:** 2024-11-25

**Authors:** Abdullah Sohail, Ammad J. Chaudhary, Muhammad Mujtaba Bhinder, Khadija Zahid, Kyle Brown

**Affiliations:** ^1^ Department of Internal Medicine University of Iowa Hospitals and Clinics Iowa City Iowa USA; ^2^ Department of Internal Medicine Henry Ford Hospital Detroit Michigan USA; ^3^ Charleston Area Medical Center West Virginia USA; ^4^ Gujranwala Medical College Gujranwala Pakistan; ^5^ Department of Internal Medicine, Gastroenterology and Hepatology University of Iowa Hospitals and Clinics Iowa City Iowa USA

**Keywords:** hepatorenal syndrome, inpatient mortality, predictors of readmission, readmission rate, resource utilization

## Abstract

**Background:**

Nationwide US data on readmission rates for patients with cirrhosis admitted with hepatorenal syndrome (HRS) is lacking. We reviewed 30‐day readmission rates after HRS‐related hospitalizations, the associated predictors of readmissions, and their impact on resource utilization and mortality in the United States.

**Methods:**

We identified all adults admitted with HRS between 2016 and 2019 using the Nationwide Readmission database of the Agency for Healthcare Research and Quality's Healthcare Cost and Utilization Project. The primary outcome was all‐cause 30‐day readmission rate. Secondary outcomes were inpatient mortality rate, predictors of readmission, and resource utilization.

**Results:**

We identified 245 850 hospitalizations of patients admitted for HRS in the United States from 2016 to 2019. Of these, 214 890 met the inclusion criteria. Mean age was 59.16 years, and 61.31% were males. Medicare was the most common primary payer (44.82%) followed by Medicaid (25.58%). The readmission rate was 24.6% within 30 days of discharge from index hospitalization. The most common cause of readmission was alcoholic cirrhosis with ascites (14.87%), followed by sepsis (9.32%) and unspecified hepatic failure (9%). The in‐hospital mortality rate for index hospitalization was 29.52% and 14.35% among those readmitted within 30 days. The mean length of stay (12.33 days vs. 7.15 days, *p* < 0.01) and hospitalization costs ($44 903 vs. $22 353, *p* < 0.01) were higher for index hospitalizations than readmissions.

**Conclusions:**

Our study demonstrated that all‐cause 30‐day readmission and in‐hospital mortality rates after the development of HRS were strikingly high. This warrants health policies and interventions at the institutional level, including close post‐hospital discharge follow‐up, to decrease readmission rates, improve patient outcomes, and reduce cost burden.

## 1 Introduction

Hepatorenal syndrome (HRS) is a severe complication of cirrhosis that leads to progressive kidney dysfunction with high morbidity and mortality rates [1, 2]. HRS is common among hospitalized patients with cirrhosis and ascites, with an incidence of approximately 10% [3]. In patients with decompensated cirrhosis, the likelihood of developing HRS within 1 year ranges from 8% to 20% and escalates to 40% over a period of 5 years. Furthermore, an estimated 35%–40% of patients with end‐stage liver disease (ESLD) and ascites eventually develop HRS [4]. Allegretti et al. reported a 90‐day mortality rate of 57% among patients with HRS [5].

HRS is categorized into two types based on disease severity [6]. HRS acute kidney injury (AKI), formerly known as Type 1, leads to a rapid decline in kidney function due to poor renal perfusion, marked by a significant increase in serum creatinine levels (≥ 0.3 mg/dL) within 48 h and/or urine output ≤ 0.5 mL/kg body weight for ≥ 6 h [7, 8]. This type is rapidly progressive, with median survival typically ranging from 8 to 12 weeks [9]. HRS non‐acute kidney injury (NAKI), formerly known as Type 2, is less acute and is characterized by diuretic‐resistant ascites. It is further classified into HRS acute kidney disease (AKD) and HRS chronic kidney disease (CKD), based on eGFR < 60 mL/min per 1.73 m^2^ for < 3 months or ≥ 3 months, respectively, in the absence of any other structural causes [8]. This type progresses more slowly and has a median survival of approximately 6 months [7, 9].

The economic impact of liver disease on the US healthcare system is significant, with direct costs surpassing $3.5 billion yearly, and cirrhosis represents a substantial portion of these costs [10]. Hospital readmissions, which are often preventable, markedly contribute to resource utilization. Previous studies have shown an increasing incidence of HRS hospitalizations in patients with chronic liver disease in the United States [11]. Existing studies on cirrhosis‐related readmissions have reported a 30‐day rate of 23% [12]. Additionally, HRS has been identified as an independent predictor of readmission in patients with decompensated cirrhosis [13]. In a retrospective analysis conducted by Rice et al., 30‐day readmission rates for patients admitted with HRS ranged from 26% among those with commercial insurance to 36% for those covered by Medicare [14]. Additionally, the median survival duration was 95 days for commercially insured patients compared to 33 days for those on Medicare. In contrast, another study by Jamil et al. documented a 30‐day readmission rate of 33% and a mortality rate of 36.9% among patients with HRS [1]. To our knowledge, no previous nationwide studies have explored the patient and hospital characteristics of these readmissions. This variability in reported outcomes may stem from divergent data availability, sample sizes, research methodologies, and varied healthcare settings. Consequently, our study aimed to investigate 30‐day readmission rates of HRS patients, identify the underlying causes and predictors of these readmissions, and evaluate their impact on healthcare utilization and patient survival outcomes.

## 2 Methods

### 2.1 Data Source

We performed a retrospective cohort study using the National Readmission Database (NRD) to identify all patients with HRS. Patients were hospitalized in the United States from 2016 through 2019. The NRD is part of the Healthcare Cost Utilization Project (HCUP) within the Agency of Healthcare Research and Quality and provides national‐level estimates of hospital readmissions in the United States. The NRD is also the largest publicly available readmission database in the United States, containing clinical and demographic information for all hospitalizations from participating states, which increased from 22 states in 2010 to 30 in 2019 [15]. It is designed in a stratified probability sample to represent all non‐federal acute‐care inpatient hospitalizations, including patient‐ and hospital‐level information. The hospital‐level data includes ownership, teaching status, number of beds, rural/urban location, and geographical location. Patient‐level data includes demographics such as age, sex, median income by zip code quartile, principal and secondary diagnoses at discharge, readmission rate, length of stay, procedures performed, and total hospitalization cost and charges. Discharge diagnosis and procedure codes were identified using the *International Classification of Diseases, Tenth Revision, and Clinical Modification* (ICD10‐CM) [16].

### 2.2 Study Population

**FIGURE 1:**
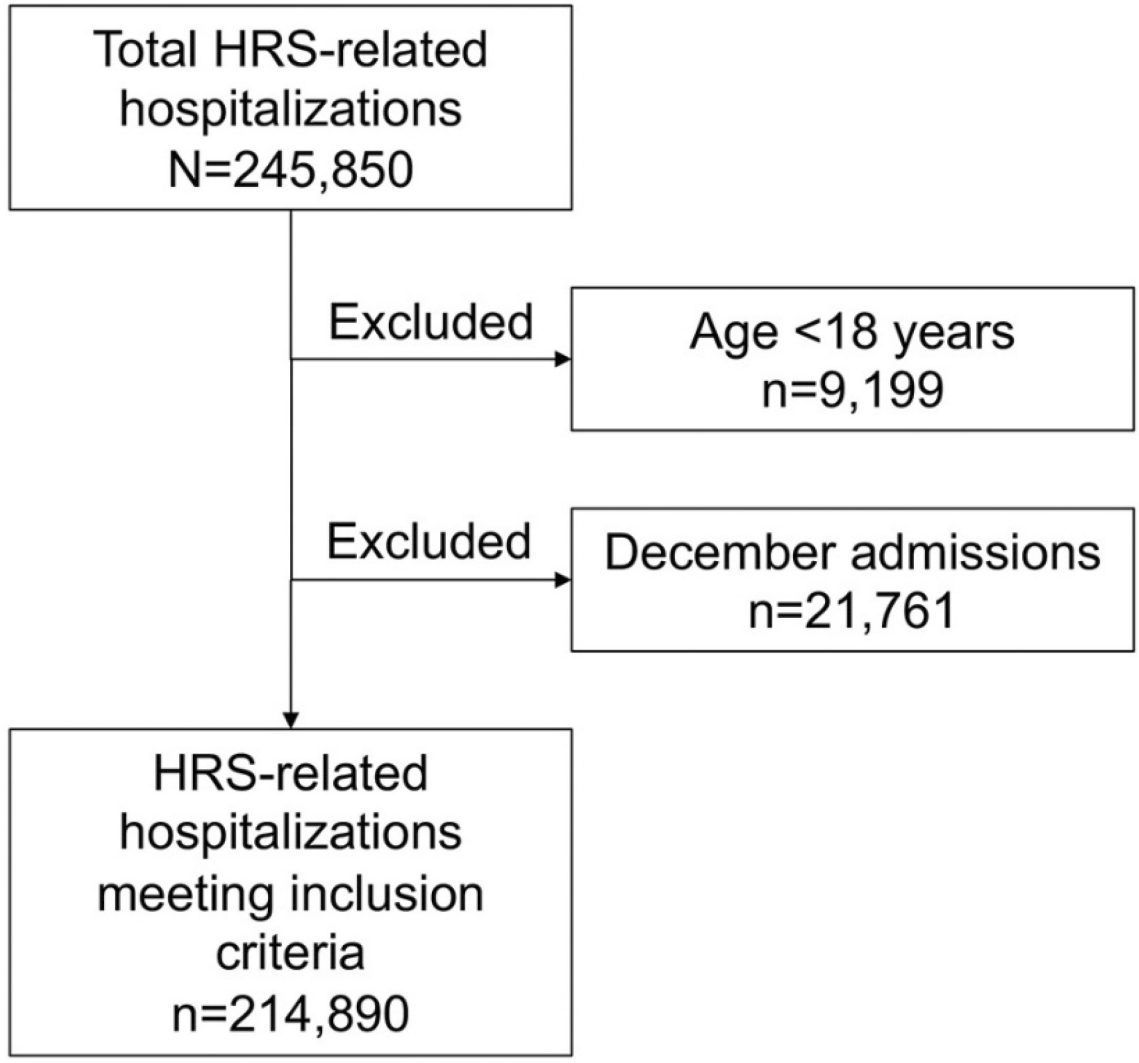
Patient selection flow diagram. HRS, hepatorenal syndrome.

We identified all patients admitted with a primary diagnosis of HRS using the ICD‐10 code (K76.7), which has been used in previously published studies (Figure 1) [17, 18]. All adult patients aged > 18 were included. Since the NRD tracks hospitalizations on a calendar‐year basis (i.e., January 1 through December 31) without linking them to the previous or following year, we excluded all index hospitalizations in December.

### 2.3 Study Outcome

The primary outcome of our study was all‐cause 30‐day readmission rates for patients admitted for HRS and the most common reasons for readmission. NRD assigns a unique database identification number to each patient. This unique number can be used to identify all admissions for each patient. Readmission was defined as any non‐traumatic admission within 30 days of index admission. We considered only the first readmission if the patient had multiple readmissions. All patients who died during index hospitalization were excluded from the study.

Secondary outcomes included all‐cause mortality for index hospitalizations and readmissions, independent predictors of readmission, resource utilization including mean length of stay, and mean hospitalization costs for index hospitalizations and readmissions. We also examined the need for intensive care unit (ICU) admission, mechanical ventilation (MV), and incidence of septic shock in patients hospitalized with HRS.

### 2.4 Patient and Hospital Characteristics

We used NRD variables to identify patient age, sex, race, median household income per zip code, primary insurance payer (Medicare, Medicaid, private, and uninsured), and residential areas (rural or urban). Hospital characteristics included hospital size based on number of beds (large, medium, and small), urban versus rural location, teaching versus non‐teaching status, and geographical location. ICD‐10 codes were used to identify all comorbid conditions, including hypertension, diabetes mellitus type 2 (T2DM), chronic obstructive pulmonary disease (COPD), nicotine dependence, and alcohol use disorder. The NRD also provides information about the total hospitalization charges that hospitals bill for each hospitalization. However, total hospitalization cost reimbursed by the primary payer was calculated using cost‐to‐charge ratio files provided by HCUP [19]. Deyo's modification of the Charlson Comorbidity Index (CCI) was used to assess the patient's comorbidity burden [20].

### 2.5 Statistical Analysis

Statistical analysis was performed using STATA (version 16.0; Stata Corp., College Station, TX). NRD design includes stratification, clustering, and weighing. STATA facilitates a detailed analysis to produce a national representation of the results, variance estimates, and *p* values. Estimates of all hospitalized patients with HRS in the United States were obtained after weighing patient‐level observations. The *χ* ^2^ test was used for categorical variables, and Student's *t* test was used for continuous variables. *p* values were two‐sided, with a statistical significance threshold of 0.05. Survival analysis was performed with time from discharge to readmission as a time variable and death as a failure. Patients were censored on the 30th day of discharge if they were alive. The unadjusted hazard ratio was calculated using univariate Cox regression analysis for predictors of readmission. We built a multivariate model from the variables to account for confounding factors with a cutoff *p* value of less than 0.2 on univariate analysis. Observations with missing values were excluded.

## 3 Results

From 2016 through 2019, we identified 245 850 patient hospitalizations related to HRS, of which 214 890 met our inclusion criteria of age ≥ 18 years and admission other than in December (Figure 1). The baseline characteristics of patients at index hospitalization and those readmitted within 30 days are shown in Table 1. The average age was slightly higher during index admission (59.16 years) than during readmission (58.09 years). The proportion of males was higher in both groups (61.31% for index admission and 60.96% for readmission). A significant majority of patients in both groups had a CCI greater than 3 (92.07% at index and 93.31% at readmission). Median income based on zip code showed no significant difference between the index and readmission groups, with the largest segment in earnings between $1 and $38 999 (29.82% vs. 30.3%, respectively, *p* = 0.13). The predominant type of insurance coverage was Medicare (44.82% for index admissions and 43.23% for readmissions), followed by Medicaid and private insurance. Notably, comorbid conditions such as hypertension, D.M., and nicotine dependence were more prevalent during readmissions, showing statistically significant increases (*p* < 0.01). In both groups, most of the patients were admitted to large urban teaching hospitals.

**TABLE 1:** Baseline demographic characteristics of HRS hospitalizations and 30‐day readmissions in the United States.

	Index admission	30‐day readmission	*p*
No. of patients (*N*)	214 890	37 266	
Mean age (years)	59.16	58.09	
Male (*N*, %)	131 749 (61.31%)	22 717 (60.96%)	0.39
Female (*N*, %)	83 141 (38.69%)	14 549 (39.04%)	0.39
Charlson Comorbidity Index			< 0.01
0	430 (0.20%)	186 (0.5%)	
1	7521 (3.5%)	75 (0.2%)	
2	9090 (4.23%)	11 (0.03%)	
3 or more	197 849 (92.07%)	34 773 (93.31%)	
Median income based on zip code			0.13
$1–38 999	64 080 (29.82%)	11 292 (30.3%)	
$39 000‐47 999	61 502 (28.62%)	10 520 (28.23%)	
$48 000–62 999	52 003 (24.2%)	9231 (24.77%)	
> $63 000	37 262 (17.34%)	6257 (16.79%)	
Insurance provider (*n*, %)			0.01
Medicare	96 314 (44.82%)	16 110 (43.23%)	
Medicaid	54 969 (25.58%)	10 584 (28.4%)	
Private	54 690 (25.45%)	9536 (25.59%)	
Other	8875 (04.13%)	1029 (2.76%)	
Comorbid conditions			
Hypertension	72 590 (33.78%)	13 785 (36.99%)	< 0.01
T2DM	37 283 (17.35%)	8072 (21.66%)	< 0.01
COPD	36 617 (17.04%)	6428 (17.25%)	0.53
Congestive heart failure	47 985 (22.33%)	7781 (20.88%)	< 0.01
Nicotine dependence	44 160 (20.55%)	9372 (25.15%)	< 0.01
Alcohol use disorder	36 961 (17.2%)	5907 (15.85%)	< 0.01
Hospital characteristics			
Hospital teaching status			0.18
Metropolitan non‐teaching	45 557 (21.20%)	7718 (20.71%)	
Metropolitan teaching	169 312 (78.79%)	29 548 (79.29%)	
Hospital bed size			0.17
Small	28 559 (13.29%)	4900 (13.15%)	
Medium	52 046 (24.22%)	8758 (23.5%)	
Large	134 263 (62.48%)	23 608 (63.35%)	
Hospital location			0.56
Rural	28 408 (13.22%)	4990 (13.39%)	
Urban	186 460 (86.77%)	32 276 (86.61%)	
Complications			
ICU	52 197 (24.29%)	4021 (10.79%)	< 0.01
MV	45 105 (20.99%)	3276 (8.79%)	< 0.01
Septic shock	25 550 (11.89%)	2471 (6.63%)	< 0.01
Mortality	63 436 (29.52%)	5348 (14.35%)	< 0.01
Resource utilization			
Length of stay (days)	12.33	7.15	< 0.01
Mean hospitalization cost	$44 903	$22 352	< 0.01

Abbreviations: COPD, chronic obstructive pulmonary disease; ICU, intensive care unit; MV, mechanical ventilation; T2DM, type 2 diabetes mellitus.

### 3.1 30‐Day All‐Cause Readmissions

**FIGURE 2:**
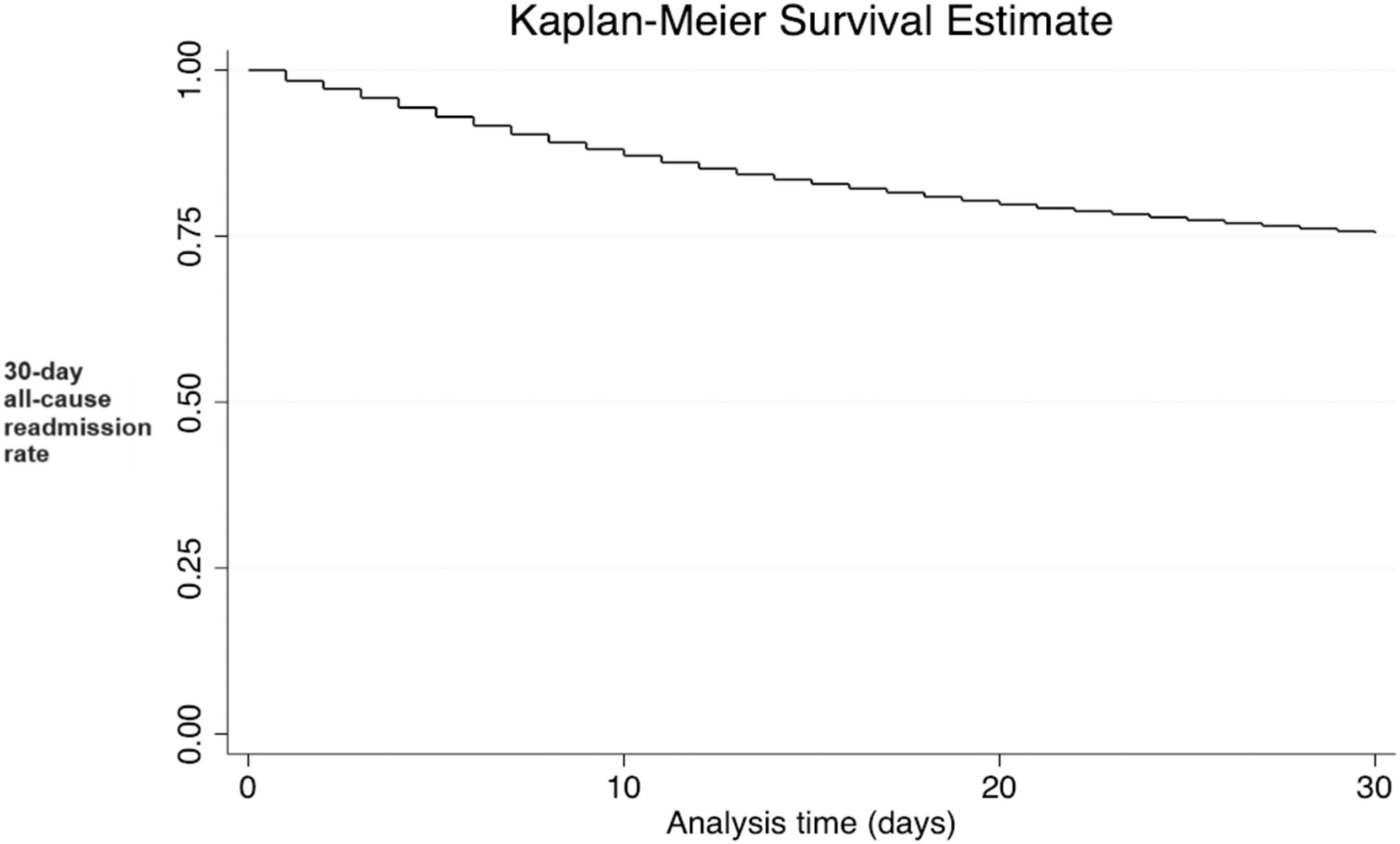
Kaplan–Meier curve for 30‐day all‐cause readmission among patients with HRS.

Of the 151 454 discharged patients, 24.60% (37 266) were readmitted within 30 days. The Kaplan–Meier curve depicted in Figure 2 shows the survival probabilities over this period. The primary cause of readmission was alcoholic cirrhosis with ascites (14.87%), followed by sepsis (9.32%), unspecified hepatic failure without coma (9%), acute renal failure (5.21%), and HRS (4.45%) as shown in Table 2.

**TABLE 2:** Most common causes of readmission for patients with HRS.

Primary diagnosis of readmission	% of patients
Alcoholic cirrhosis of the liver with ascites	14.87
Septic shock	9.32
Hepatic failure, unspecified without coma	9
Acute renal failure, unspecified	5.21
HRS	4.45

Abbreviation: HRS, hepatorenal syndrome.

### 3.2 In‐Hospital Mortality Rates Among Admissions and Readmissions

A total of 63 436 (29.52%) patients admitted with HRS died during the index hospitalization. Among the patients readmitted within 30 days who had HRS during their initial hospitalization, 5348 (14.35%) in‐hospital deaths were recorded.

### 3.3 Morbidity Among Readmitted Patients

During index hospitalization, 24.29% of patients required ICU‐level care, which was significantly higher than during the 30‐day readmission period (10.79%, *p* < 0.01). Need for MV was 20.99% at index admission and 8.79% at readmission (*p* < 0.01). Furthermore, the incidence of septic shock was significantly higher during index hospital stay (11.89%) than during 30‐day readmission (6.63%, *p* < 0.01).

### 3.4 Resource Utilization During Readmission

The mean length of stay for index hospitalization was greater than that for 30‐day readmission (12.33 days vs. 7.15 days, *p* < 0.01). The mean total hospitalization cost for index hospitalizations was $44 903, while hospitalization costs for readmission were $22 352. The 30‐day readmissions resulted in an additional 266 551 hospital days, with an additional $415 million in extra inpatient charges.

### 3.5 Independent Predictors of 30‐Day Readmissions

Independent predictors of readmission after hospitalization for HRS were Medicaid insurance, T2DM, hypertension, nicotine dependence, and alcohol use disorder. The remaining variables considered in the model did not significantly impact readmission prediction statistically. Table 3 presents factors influencing 30‐day readmissions as determined through multivariate Cox regression analysis, which considered both patient demographics and hospital characteristics.

**TABLE 3:** Independent predictors of 30‐day readmission for patients initially admitted with HRS.

	Adjusted HR (95% CI)	*p*
Female	1.02 (0.98–1.06)	
Mean age (years)	0.99 (0.92–0.99)	< 0.01
Charlson Comorbidity Index		
1	Reference	
2	0.92 (0.59–1.49)	0.73
3 or more	0.91 (0.47–1.44)	0.68
Median income based on zip code		
$1–38 999	Reference	
$39 000–47 999	0.93 (0.89–0.98)	< 0.01
$48 000–62 999	0.94 (0.90–0.99)	0.02
>$63 000	0.91 (0.87–0.96)	< 0.01
Insurance provider		
Medicare	Reference	
Medicaid	1.25 (1.20–1.31)	< 0.01
Private	1.04 (0 0.99–1.09)	0.05
Others	0.91 (0.81–1.02)	0.09
Comorbidities		
T2DM	1.26 (1.20–1.32)	< 0.01
Hypertension	1.13 (1.08–1.17)	< 0.01
Congestive heart failure	0.94 (0.90–0.98)	< 0.01
COPD	1.02 (0.97–1.07)	0.31
Nicotine dependence	1.09 (1.04–1.14)	< 0.01
Alcohol use disorder	1.05 (1.01–1.10)	0.01
Hospital teaching status		
Non‐teaching	Reference	
Teaching	1.03 (0.98–1.07)	0.2
Hospital size		
Small	Reference	
Medium	1.01 (0.94–1.08)	0.76
Large	1.02 (0.97–1.09)	0.33
Hospital region		
Rural	Reference	
Urban	0.98 (0.93–1.03)	0.57
Complications		
ICU	0.69 (0.65–0.74)	< 0.01
MV	0.69 (0.64–0.73)	< 0.01
Septic shock	0.73 (0.68–0.79)	< 0.01

Abbreviations: CI, confidence interval; COPD, chronic obstructive pulmonary disease; HR, hazard ratio; ICU, intensive care unit; MV, mechanical ventilation; T2DM, type 2 diabetes mellitus.

## 4 Discussion

In this large nationwide study conducted from 2016 through 2019, we identified 214 890 HRS‐related hospitalizations that met our inclusion criteria. Approximately one in every four patients with HRS who survived the index hospitalization was readmitted within 30 days of discharge. The most common cause of readmission was alcoholic cirrhosis with ascites, followed by sepsis and hepatic failure. Additionally, our study identified distinct patient and hospitalization characteristics that correlated with an increased risk of readmission. Multivariate Cox regression analysis identified Medicaid insurance, T2DM, hypertension, nicotine dependence, and alcohol use disorder as independent predictors of 30‐day readmissions. The in‐hospital mortality rate was 29.52% for initial admission and 14.35% for readmitted patients. Requirements for ICU and MV and the incidence of septic shock were significantly higher during initial admission. Additionally, mean length of stay and hospitalization costs were greater for initial admissions than readmissions, contributing to substantial resource utilization.

Our results are in accordance with previous studies regarding patient characteristics and demographics. Rice et al. performed a retrospective analysis of 1845 HRS patients and reported a male predominance in both privately insured (63.0%) and Medicare (57.9%) populations. However, our study observed a slightly older average age of 59.16 years at index admission, compared to 54.1 years. The prevalence of comorbid conditions, as indicated by a CCI greater than 3 in a significant majority of our patient cohort, points to a high burden of comorbidity similar to that reported by Rice et al. (CCI of 6.2 for commercial and 7.9 for Medicare) [14]. Notably, our study highlighted an increased prevalence of specific comorbidities such as hypertension, T2DM, and nicotine dependence upon readmission, highlighting the complex health profile of patients readmitted with HRS. These findings underline the significant impact of demographic and comorbid factors on clinical management and outcomes of HRS, emphasizing the need for targeted interventions in these high‐risk groups.

Only a limited number of studies have explored the 30‐day readmission rates of patients with HRS. Rice et al. reported a 30‐day readmission rate of 26% in commercially insured patients and 36% in Medicare patients [14]. Our nationwide analysis found a 30‐day readmission rate of 24.60% among discharged patients with HRS, which is marginally lower than that previously reported. The differences in study design, sample size, and healthcare systems can explain this observed variability. In addition, the study by Rice et al. had age limitations (commercially insured patients aged 18–64 and Medicare patients aged 65 and older), which could have resulted in a higher readmission rate in their population cohort. Our study is the first in the United States to explore the most common reasons for these readmissions and their independent predictors. The primary causes of readmission were alcoholic cirrhosis with ascites, sepsis, and hepatic failure, among others, highlighting the medical complexities faced by these patients. A retrospective study of 1429 patients with alcoholic cirrhosis identified that complications such as HRS significantly increased morbidity and mortality, explaining the recurrent readmissions [21]. Although HRS can occur across all forms of cirrhosis, its prevalence is notably higher among patients with alcoholic cirrhosis, which could explain the recurrent readmissions observed [22]. In addition, our study identified several independent predictors of readmission, such as Medicaid insurance, T2DM, hypertension, nicotine dependence, and alcohol use disorder. This detailed insight emphasizes the need for targeted healthcare interventions and tailored post‐discharge care plans to mitigate these risk factors, potentially reducing readmission rates.

Our nationwide study delineated the differential resource utilization inherent to index hospitalizations versus 30‐day readmissions. Notably, the mean length of stay for initial admissions (12.33 days) significantly exceeded that of readmissions (7.15 days, *p* < 0.01). Likewise, index hospitalization costs averaged $44 903, nearly double the $22 352 for readmission. In contrast, Rice et al. outlined a 90‐day post‐discharge cost accumulation, identifying an increase in mean healthcare costs of up to $157 665 for commercially insured patients and $48 322 for Medicare beneficiaries. These variations may be attributed to their extended timeframe and the inclusion of diverse insurance models, which diverge from the inpatient cost analysis. Our findings emphasize the acute financial and operational impact of readmissions, accounting for an additional 266 551 hospital days and $415 million in cost. While the Rice study provides insights into extended healthcare expenses, our nationwide data underline the need for improved discharge protocols to curtail readmissions, presenting a pivotal opportunity for enhancing healthcare efficiency and cost containment. Institutional protocols for the treatment of HRS, including the use of vasopressors and albumin, play a crucial role in reducing mortality and potentially lowering readmission rates. As highlighted by recent studies, evidence‐based protocols for the diagnosis and treatment of HRS have been independently associated with improved survival outcomes, even when reducing the total doses of terlipressin and albumin used. Incorporating such protocols may help optimize treatment strategies, reduce healthcare costs, and improve patient outcomes, as suggested by Terres et al. [23].

Our study has several inherent limitations. The retrospective database, primarily serving administrative and billing purposes, lacks detailed clinical information, crucially omitting laboratory results that could indicate the severity of HRS. In addition, the absence of data on medications taken by patients limits our understanding of the impact of treatment and patient compliance. The inpatient‐only focus of the database excluded outpatient follow‐ups, introducing a potential selection bias. The inability to establish causality, combined with the specific context of the US healthcare system, affects the generalizability of our findings. Furthermore, the time‐bound nature of the data restricts the follow‐up period to within 1 calendar year, which may not capture the full trajectory of patient outcomes, particularly for those discharged later in the year. External factors such as community resources and social determinants play a significant role in readmission rates and are not accounted for. These constraints necessitate a cautious approach in interpreting our results and highlight the need for more comprehensive research to fill these gaps and to better understand the complex dynamics surrounding HRS readmissions. Liver‐specific scores such as MELD‐Na are crucial for predicting outcomes in patients with HRS. Although these analyses were not included in our study, we acknowledge their relevance in predicting readmissions and mortality. Studies have demonstrated that scores like the Child‐Turcotte‐Pugh (CTP) and MELD‐Na play a significant role in prognosticating mortality, guiding treatment decisions, and resource allocation for HRS patients. Incorporating these scores into future analyses could help target interventions more effectively [24]. It is important to acknowledge that many readmissions for patients with advanced cirrhosis and HRS may be unavoidable without a curative liver transplant. While supportive management strategies, such as adherence to low salt diets, alcohol abstinence, SBP prophylaxis, and encephalopathy treatments, are crucial, they may not significantly alter long‐term outcomes in this population. As such, our findings should be interpreted with the understanding that readmission rates may reflect the terminal nature of these patients' conditions.

Regardless of the above‐mentioned limitations, we believe our study is the first in the United States to explore the reasons, predictors, and impact of HRS readmissions on the United States healthcare system. In summary, our comprehensive national study revealed a high rate of hospital readmissions among patients with HRS, emphasizing the significant challenge it poses to the healthcare system. This study identified key comorbidities and socioeconomic elements contributing to these readmissions, highlighting their influence on patient outcomes. Our findings also indicate substantial use of healthcare resources for managing HRS, reinforcing the need for better discharge and post‐hospitalization strategies. Addressing these factors through policy changes and improving clinical management can potentially enhance patient care and reduce the healthcare burden of HRS.

## Conflicts of Interest

The authors declare no conflicts of interest.

## Data Availability

The data analyzed in this study is publicly available.
